# An impact model to understand and improve work-life balance in early-career researchers in radiation oncology

**DOI:** 10.1016/j.ctro.2022.09.006

**Published:** 2022-09-26

**Authors:** Carina Pittens, Jennifer Dhont, Steven Petit, Ludwig Dubois, Pierfrancesco Franco, Laura Mullaney, Marianne Aznar, Violet Petit-Steeghs, Jenny Bertholet

**Affiliations:** a Athena Institute, VU University Amsterdam, Amsterdam, the Netherlands; b Department of Medical Physics, Institut Jules Bordet, Université Libre de Bruxelles (ULB), Brussels, Belgium; c European Society for Radiotherapy & Oncology (ESTRO) Young Committee, Brussels, Belgium; d Department of Radiotherapy, Erasmus MC Cancer Institute, Rotterdam, the Netherlands; e The M-Lab, Department of Precision Medicine, GROW – School for Oncology and Reproduction, Maastricht University, Maastricht, the Netherlands; f Department of Translational Medicine (DIMET), University of Eastern Piedmont, Novara, Italy; g Applied Radiation Therapy Trinity Research Group, Discipline of Radiation Therapy, School of Medicine, Trinity College Dublin, Ireland; h Division of Cancer Sciences, Faculty of Biology, Medicine and Health, The University of Manchester, The Christie NHS Foundation Trust, Manchester, UK; i Nuffield Department of Population Health, University of Oxford, Oxford, UK; j Erasmus School of Health Policy and Management, Erasmus University Rotterdam, Rotterdam, the Netherlands; k Division of Medical Radiation Physics and Department of Radiation Oncology, Inselspital, Bern University Hospital and University of Bern, Bern, Switzerland

**Keywords:** Early-career, Working conditions, Mental health, Qualitative research

## Abstract

**Purpose:**

The COVID-19 pandemic had a substantial effect on mental health and work productivity of early-career researchers working in Radiation Oncology (RO). However, the underlying mechanisms of these effects are unclear. The aim of the current qualitative study was therefore to achieve a better understanding of how these effects arose and could be managed in the future.

**Methods:**

This study was conducted jointly by RO and qualitative health researchers. Data was collected in four online Focus Groups with 6–11 RO researchers (total N = 31) working in Europe. The transcripts were analysed through a qualitative cross-impact analysis.

**Results:**

Causal relations were identified between seventeen variables that depict the impact of disrupted working conditions. Mental health and work productivity were indeed the most important affected variables, but relations between variables towards these impacts were complex. Relations could either be positive or negative and direct or indirect, leading to a cascade of interrelated events which are highly personal and could change over time. We developed the model ‘impact of disrupted working conditions’ depicting the identified variables and their relations, to allow more individual assessment and personalised solutions.

**Conclusion:**

The impacts of disrupted working conditions on RO researchers varied due to the complexity of interrelated variables. Consequently, collective actions are not sufficient, and a more personal approach is needed. Our impact model is recommended to help guide conversations and reflections with the aim of improving work/life balance. The participants showed high levels of personal responsibility towards their own mental health and work productivity. Although being an individual issue, a collective responsibility in developing such approaches is key due to the dependency on organizational variables.

## Highlights

• Disrupted working conditions, such as during COVID-19, substantially impacted RO researchers.
• The impact varied due to the complexity of interrelated variables.
• There is a collective responsibility to find solutions that adequately address individual needs.
• We developed an impact model for disrupted working conditions.
• The model can be used in conversations and reflections.

## 1 Introduction

By early January 2021, less than one year after the World Health Organizatioan (WHO) declared Coronavirus Disease 2019 (COVID-19) a pandemic, the WHO has reported over ninety million confirmed cases and over two million COVID-19 deaths [1]. Several nationwide surveys studying the effects of COVID-19 among the general population have shown increased levels of psychological strain including stress, anxiety, depression, insomnia, post-traumatic stress disorder and other common mental health disorders [2], [3], [4], [5]. The impact on healthcare professionals was particularly critical and complex. While some studies report higher levels of general anxiety, depressive symptoms and stress compared to the general population, other reports indicate lower traumatisation of healthcare personnel providing direct care for patients with COVID-19 [6], [7], [8], [9], [10]. Nevertheless, most studies conclude that the prolonged burden of medical staff might have significant long-term effects, with large percentages meeting the criteria for burnout [11], [12], [13], [14], [15].

A survey distributed among Radiation Oncology (RO) researchers during the first COVID-19 peak, gathering 543 responses, showed a clear negative impact on perceived productivity and mental health [16]. Of the respondents, 71.2 % were feeling less productive and 58 % perceived some level of guilt associated to their productivity. Compared to normative data, higher levels of both anxiety and depressive symptoms were recorded for the 335 respondents who filled-in the accompanying Hospital Anxiety and Depression Scale (HADS) [17], [18]. Depressive symptoms were associated with working on location – as opposed to working (full or part-time) at home - while symptoms of anxiety were negatively correlated to years of research experience, meaning early-career researchers were at higher risk for anxiety symptoms. The study demonstrated the scale of the impact on mental health and productivity, but only offered speculations regarding variables that caused these impacts. Further, no other possible impacts in addition to productivity and mental health were investigated, and the study could not provide targeted solutions or possible actions. In line, other studies, investigating disrupted working conditions in health care, mainly focussed on identifying disrupting working conditions and their impacts; paying limited attention to underlying mechanisms and how variations in impacts could be explained [19], [20], [21], [22], [23].

The aim of this study was therefore to gain a deeper understanding of the impact of disrupted working conditions on early-career RO professionals involved in research, with the purpose to identify solutions for the RO community. We believe that the insights of this study are valuable for developing future occupational measures in case of disrupted working conditions, targeting both employers and employees within the RO community.

## 2 Methods

This qualitative research study, consisting of four online focus groups (oFGs), was a collaboration between researchers in the field of RO (JD, JB, SP, LD, PF, LM, MA) and experts in qualitative research on multi-stakeholder involvement regarding complex societal issues (CP, VPS). The qualitative experts led the design and analysis and facilitated the oFGs, thereby safeguarding the quality of the research methodology. Qualitative research is the method of choice to gain a deeper understanding of people’s experiences and perspectives [24], [25], [26], [27]. By stimulating discussion among participants with common traits or experiences, focus groups aim to generate a broad array of shared and individual experiences and in-depth perspectives [26].

### 2.1 Participants

Early-career RO professionals involved in clinical or academic research working in Europe with less than ten years of research experience were invited to participate in the oFGs. The latter criterion was instated because the impact on mental health was found to be especially high for this group of RO researchers [16]. Recruitment was carried out through the European Society for Radiotherapy and Oncology (ESTRO) newsletter, social media and personal networks of part of the authors. Convenience sampling was applied, but efforts were made to balance gender, country of residence and RO subdiscipline per oFG. In total 31 participants volunteered and were available to participate in an oFG (6–11 per oFG). An overview of participant characteristics can be found in Table 1. All oFG participants gave written informed consent to participate in this study. The research complies with the Dutch Code of Ethics for Research in the Social and Behavioural Sciences involving Human Participants (VCWE, 2016).

**Table 1:** Socio-demographical and work-related variables of the 31 participants in the oFGs.

	Total (N = 31)
**Age (Mean (SD))**	32 (4)
**Gender (N (%))**	
Female	25 (81 %)
Male	6 (19 %)
**Country (N (%))**	
Albania	1 (3 %)
Belgium	1 (3 %)
Denmark	2 (6 %)
Germany	1 (3 %)
Ireland	1 (3 %)
Italy	4 (13 %)
Netherlands	7 (23 %)
Romania	1 (3 %)
Slovenia	2 (6 %)
Spain	1 (3 %)
Switzerland	2 (6 %)
United Kingdom	8 (26 %)
**Position (N (%))**	
PhD student Medical Physics Radiation Oncology Radiation Technology Biology/Pharmacology	9 (29 %)3 (10 %)4 (13 %)1 (3 %)1 (3 %)
Post-Doctoral researcher Medical Physics Radiation Oncology Biology/Pharmacology	6 (19 %)4 (13 %)1 (3 %)1 (3 %)
Assistant Professor Medical Physics Radiation Technology	2 (6 %)1 (3 %)1 (3 %)
No current academic position Medical Physics Radiation Oncology Radiation Technology	14 (45 %)4 (13 %) 8 (26 %)2 (6 %)
**Work situation (N (%))**	
Full-time at home	11 (35 %)
Full-time on location	10 (32 %)
Part-time on location	10 (32 %)

### 2.2 Data collection

**Fig. 1:**
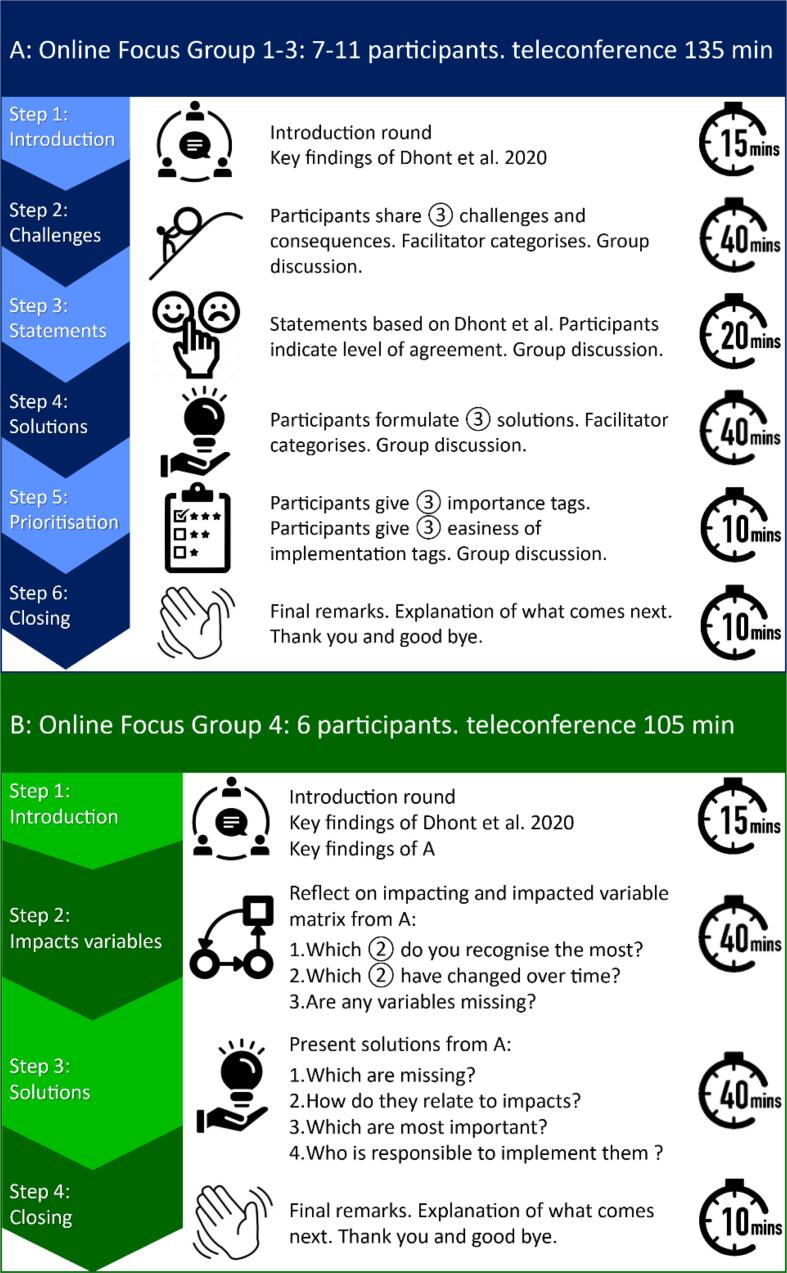
Design of oFG 1–3 (A) and the validation oFG 4 (B), including the aims and methods of the different steps, and the applications used within each step.

Data was collected in two-steps. First, three sequential oFGs (Oct. – Nov. ‘20) were conducted to: (1) identify all variables that caused, enabled, or exacerbated impacts in the context of disrupted working conditions, and (2) formulate solutions that could positively address the identified negative impacts. As illustrated in Fig. 1A, these points were addressed in six steps, each with a specific aim, strategy and application to support data extraction [28], [29]. Secondly, a fourth oFG was conducted (Apr.’21) to: (1) validate the results of the first three oFGs and ensure data saturation was reached, and (2) formulate and prioritise solutions to address the identified negative impacts. As illustrated in Fig. 1B, these elements were further divided in four steps. All oFGs were led by an expert in focus group research (CP) and co-facilitated by one RO professional (JD and/or JB) who also shared their own experience (not included in the 31 participants).

### 2.3 Analysis

The oFGs were recorded and transcribed verbatim. Subsequently, transcripts were analysed by the qualitative research experts (VPS, CP), through a multi-step qualitative cross-impact analysis (CIA) using the analysis software Atlas – version Ti.8 (Berlin, Germany). In the CIA, ‘influential variables’ that have an ‘impact’ on ‘affected variables’ within a system are identified and classified^3^ [30], [31]. First, deductive analysis was used to categorise the data in three main domains - (1) *work sphere,* (2) *personal*/*social sphere,* and (3) *spatial/physical sphere*, where the latter refers to the physical environment or space within which work is performed [32]. Second, variables and their direct and indirect relations within these main domains were identified in three steps: (1) *open coding* (identifying, categorising and describing variables), (2) *axial coding* (identifying key variables), and (3) *selective coding* (mapping the direct and indirect relations among variables; creating cross-impact tables) [33]. Based on the CIA, personas were created to illustrate and contextualise the diversity of variables and their impacting relations [34], [35], [36], [37]. A persona is a representation of the experiences and behaviours of a group of people, often synthesised from qualitatively collected data.

## 3 Results

### 3.1 An impact model for disrupted working conditions

In total, eight influential variables and nine affected variables were identified (Table 2). Of the affected variables, *mental health* and *social life* were identified in the personal/social sphere, while all the others belonged to the work sphere, no variables belonging to the spatial /physical sphere were identified.

**Table 2:** Definitions of identified influential and affected variables.

**Influential variable**	**Definition**
Job description	Profession/subspecialty (radiation oncologist, medical physicist, RTT, biologist), appointment (PhD student, professor, head of department) and main duties (clinical research, education)
Supervision style	The type of support received from supervisor(s); the communication with supervisor(s) and how supervisor(s) managed the situation
Job experience	Level of seniority
Family situation	Presence of a partner, relatives and/or children at home or whether family lived far away.
Living situation	Living environment related to space (in and outside the home)
COVID-19 severity	Local severity of the COVID-19 pandemic
Weather	The weather and seasonal climate at a specific time and place
National measures	The measures a country or region took in reaction to the number of COVID-19 infections

**Affected variable**	**Definition**

Productivity	The amount of work the RO researchers felt they were doing, mostly compared to the period before the COVID-19 pandemic
Work quality	The self-perceived quality of the work the RO researchers were doing
Workload	The self-perceived amount of work the RO researchers had to perform
Type of work	The specific tasks the RO researcher had to perform (which can refer to e.g. clinical duties, research lab work, data science, etc.)
Accountability	The way employees are responsible and account for their actions, behaviours and decisions, and the way possible accountability structures are organised by the supervisor/superior.
Professional contacts	The amount and type of contact with colleagues from the same or other institutions
Work location	Where participants were working during the pandemic (e.g. small student room, home with garden, office at work.)
Mental health	The self-perceived mental health state of the RO professional
Social life	The amount and type of contact with friends and/or family outside a professional context

**Fig. 2:**
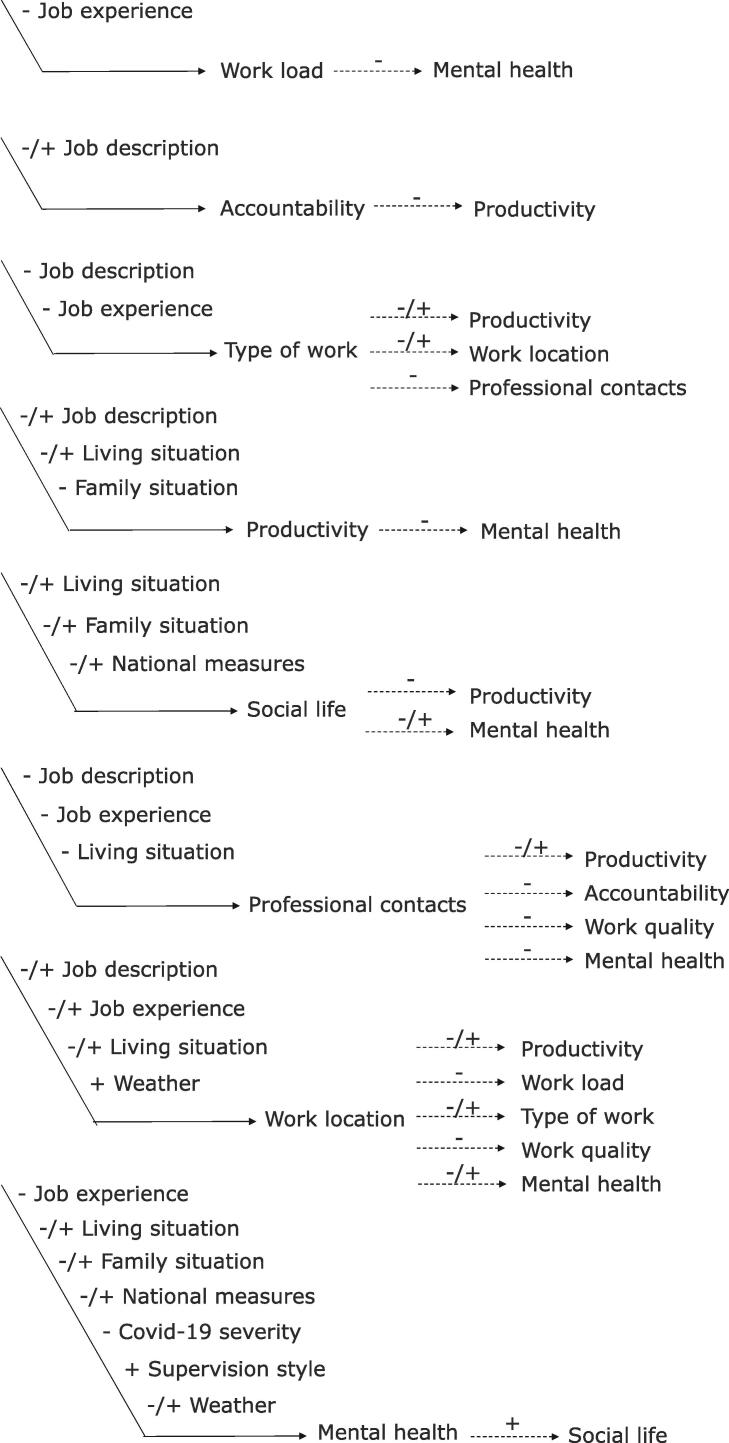
The impact model for disrupted working conditions. Direct relations are indicated with a solid arrow and indirect relations are indicated with a dashed arrow. Relations were either identified as positive (+), negative (-) or either (-/+).

Causal relations were identified between the influential and affected variables (direct relations), but also between affected variables (indirect relations), illustrated in an impact *model for disrupted working conditions* shown in Fig. 2. For each RO professional, different combinations of variables and relations could be present. Therefore, the impact model will differ by individual and depends on their personal experiences and contexts.

During the oFGs, most of the direct relations mentioned were initiated from the influential variables *job profile, research experience* and *living situation*, and were most often directed towards the affected variables *productivity*, *mental health,* and *work location* (Fig. 2). In the indirect relations, *work location* and *type of work* were most often cited as intermediary variables, with *productivity* and *mental health* most often cited as final affected variables. For example, it was often mentioned that working from home (*work location*) had a negative impact on *productivity,* as can also be seen in illustrative quote 1 in Supplementary Materials.

Both the direct and indirect relations could be positive (e.g. improving the related effect), negative (e.g. worsening the related effect), or either (e.g. improving the related affected variable for some participant(s) and worsen for other(s)). To illustrate, for one person the *job profile* meant primarily working in the clinic during the COVID-19 waves, resulting in less time for research, whilst for another the *job profile* meant working solely from home with more time available for research (quote 2, Supplementary Materials). Overall, mostly negative relations were articulated, from reduced *professional contacts* towards reduced *quality of work, productivity,* and *mental health* (quote 3, Supplementary Materials).

### 3.2 Time and context dependency

The way direct and indirect relations were perceived by the participants was also time and context dependent, decreasing, increasing, or fluctuating over time. Changes in relations were most strongly related to the changes in the causes *weather*, *COVID-19 severity*, (facilities in) *work location*, and *National measures*, which in turn changed their related effects. The perception of *mental health* also changed over time for some participants, mainly due to changes in the *COVID-19 severity*, the changing *weather* over the seasons and the changes in *National measures* (quote 4, Supplementary Materials).

### 3.3 Personas

To illustrate how influential and affected variables could differ across individuals, four personas are presented in Table 3. These personas are fictional but represent examples of the RO researchers that were consulted. They illustrate how certain variables can have different (positive “+” / negative “-”) impacts in the more comprehensive context of an individual, and thus how the impact model differs from individual to individual.

**Table 3:** Personas of Radiation Oncology researchers.

**Alex**	**Jessie**

*Under normal circumstances, Alex works primarily in the clinic****(job profile)***. *During the pandemic Alex had to work full-time in the clinic****(type of work)****resulting in less time for research****(productivity -)***. *Only junior colleagues were asked to work at the clinic which contributed to increased workload****(workload -)***. *Since Alex worked on location, it was still possible to see and speak to colleagues regularly****(professional contacts + )***. *Alex’s family lives in a different region****(family situation)****and they were not able to visit each other for long periods****(social contacts -****)****,****causing feelings of loneliness****(mental health -)***. *Moreover, due to clinical work, Alex was afraid to infect friends and colleagues****(mental health -)***. *This changed over time and in between waves****(COVID-19 severity)****; more protection material became available and safety protocols were designed****(National measures)***.	*Jessie has both clinical and research duties****(job profile)****, and lives alone****(living situation)***. *During the pandemic Jessie worked mainly from home****(work location)***. *In between the waves, Jessie was able to visit their work location regularly. In the beginning, Jessie had trouble finding a good routine: to start and end the working day and to find an appropriate work/life balance****(mental health -)***. *Consequently, productivity was very high****(productivity + )****, due to the fear of underperforming when compared to other colleagues. This resulted in increased stress****(mental health -)***. *Jessie’s supervisor also had a tough time and had less contact with Jessie to guide and discuss these issues****(accountability -)***. *Moreover, when the pandemic continued, and the winter started****(weather)****the loneliness became a big issue****(mental health -)***.
**Frankie**	**Sam**

*Frankie is a single****(family situation)****PhD student****(job profile)****who lives in a small dorm****(living situation)***. *During the pandemic, Frankie worked entirely from his small room****(work location -)****which at times felt suffocating, lonely and boring****(mental health -)***. *With no other commitments, Frankie had a lot of time to work on the PhD****(productivity + )***. *However, relatively limited experience****(job experience)****and the need to collaborate with colleagues, not available due to other duties****(professional contacts -)****, also resulted in reduced productivity at times****(productivity -)***. *It was also much more difficult to reach out to colleagues for a simple question or a brainstorm****(work quality -)***. *Frankie did notice that their social life changed over time. In between waves****(COVID-19 severity)****, social contact was more or less restored and Frankie found ways to be in contact digitally****(social life + )****, which helped prevent boredom and feeling uninspired****(mental health + )***.	*Sam works primarily as researcher****(job profile)****and has two children****(family situation)****. During the pandemic, Sam worked from home****(work location)****, and schools and day-care closed multiple times due to COVID-19 measures****(National measures)****. Sam was not able to work much in these times****(productivity -)****. This gave rise to feelings of guilt, in part towards colleagues who were working more than usual****(mental health -)***. *Sam’s supervisor was very understanding****(supervision style)****, which helped to put the feelings of guilt into perspective****(mental health + )***. *Moreover, at the start of the pandemic, not all facilities were in place to work from home and WIFI connection was often unstable****(work location -)****, making it impossible to do complex analysis****(productivity -)***. *On the positive side, Sam was able to spend much more time with family****(social contacts + )****increasing feelings of happiness****(mental health + )***.

### 3.5 Needs and solutions to address the experienced challenges

Participants were asked what they needed to overcome the experienced negative impacts of the disrupted working conditions. Interestingly, the participants did not articulate concrete solutions, but mentioned more general requirements, which could be categorised in four topics given in Table 4.

**Table 4:** Needs and possible solutions articulated by the oFG participants.

Need	Solution
Mental health support	(Low threshold) availability of (professional) support for mental health issues, on a voluntary basis.
Work Facilities	Availability of adequate technical equipment and access to data from home.
Adequate supervision	This particularly included assistance in scheduling work and building a routine. It was expressed that participants hoped basic guidelines would be formulated – from higher institutions (like ESTRO) - on adequate supervision.
Professional contacts	Ways to allow spontaneous chats and brainstorms.

Further, RO researchers expressed that they feel it was their own responsibility to identify and implement solutions for their experienced challenges. This was particularly relevant for the supports that related to better mental health or work routine (quote 5 and 6, Supplementary Materials). Moreover, some of the aforementioned needs were already addressed during the pandemic through global measures, e.g. work facilities to work remotely (proper VPN connection, access to Software), and other needs that were partly solved with the relaxation of COVID-19 National measures, such as more contacts with colleagues (both physically and digitally).

## 4 Discussion

We carried out the current study based on the results of a previous quantitative survey, which showed a substantial impact of the COVID-19 pandemic on the work productivity and mental health of RO researchers, especially at an early-career stage [16]. Through this qualitative study, we obtained a deeper understanding of the underlying mechanisms by which the consequences of disrupted working conditions (exemplified by the pandemic) arise and could be mitigated. These insights have led to the development of the *impact model for disrupted working conditions*. In line with the outcomes of the earlier questionnaire [16], the model shows the most impacts towards mental health and work productivity. However, the causal relations are complex. The model does not only visualise the diverse and dynamic interaction between identified variables (for instance relations could either be positive or negative and direct or indirect), but also shows that the way RO researchers are affected by these variables differs on an individual basis. In our results, we already stressed that the affected variables work location, productivity and mental health came forward most often. However, each of the identified influential variables (e.g. *job profile, living situation*) can be different per researcher (e.g. *PhD student, MD resident, RO professional with small children*) - especially in the multi-disciplinary and diverse field of RO. As a result, highly personal and variable situations arose which we illustrated via the personas.

Defining and prioritising concrete solutions to benefit all researchers in RO is therefore not straightforward. Although mainly collective measures have been implemented in organisations in relation to COVID-19 which is considered a communal issue, this study shows that collective actions are expected to be insufficient and sometimes counterproductive. A need for both institutional and more personal – informal – support was articulated. This is in line with insights of Van der Goot and colleagues – who investigated impacts of frontline workers in healthcare organizations during the COVID-19 pandemic [38]. They emphasized the need for relatedness support, where feeling of connectedness with colleagues (and others) is vital in coping with emotionally demanding situations. This type of support cannot (solely) be addressed in the institutional setting but is also part of the personal and social environment of RO researchers.

This need for tailored solutions – also addressing the social environment - may explain why the participants of the oFGs often felt personally responsible to find solutions, despite the earlier survey showing a significant correlation between the presence of institutional support and both lower anxiety and depressive symptoms [16]. These overall feelings of personal responsibility for one’s health follow a general trend in society of health individualisation, where well-intended initiatives such as modalities for active patient participation can also increase pressure on the individual [39], [40], [41], [42]. At the same time, it was often mentioned during the oFGs that supervisors or superiors were willing to be supportive and help, but did not know how, in this unique situation of a global pandemic.

Despite the large variety in situations and needs, we were able to formulate the following recommendations:

1. The impact model for disrupted working conditions (Fig. 2) can be used to gain insight into the complex dynamics between variables but can also serve as a template or topic list for supervisors/superiors. It can be a starting point for conversation and reflection with RO researchers about their situations, and to draw a path towards personalised solutions in case of disrupted and evolving working conditions. Management, Human Resource and/or Occupational Health personnel could also use the template to steer discussions around occupational health and well-being. The model template is available in editable format in Supplementary Materials.
2. For the impact model to be used in an appropriate way, communication with researchers and those in leadership positions remains crucial, where the first party is encouraged to verbalise their specific needs, and the latter to be open to personalised solutions and flexibility. Starting these discussions should be a low-threshold action for all team members. As such, different perspectives between groups with different leadership levels, as observed by Bertholet et al. [43], can be combined to focus on constructive solutions where they are needed. This communication should be organised through team reflection, making it a collective responsibility to find solutions that adequately address individual needs. An example of such a process was recently reported by Hernandez et al. [44]
3. Societies and institutions are encouraged to integrate adaptive skills in leadership training, to increase the supervisors’ capacity to adapt in a dynamic context where employees have different contexts and needs [45], [46], [47]. An adaptive leadership style implies solving problems by engaging others, which follows our second recommendation concerning team reflections and activate engagement on all levels.

By questioning the participants on the situation at the beginning of the pandemic and at the time of the oFGs, time-dependency of the impacts and context also became apparent. The latter reflects on findings by Slotman et al. [48], who distributed two surveys to Heads of Department and reported a decrease in telemedicine, an increased use of protective equipment, but also less social distancing from May 2020 to February 2021 in RO centres throughout Europe. Heads of Department were also increasingly concerned about their employee's well-being, burnout and work/life balance and concerned about creating flexible work arrangements. These concerns were already highlighted in our previous survey, but the current study provides recommendations and solutions to address the identified issues. Moreover, our study emphasises the importance of ongoing communication about RO researchers needs. Interestingly, no variable belonging to the spatial/physical sphere was identified. This may be due to the evolution in time where the importance of the spatial/physical sphere was more relevant in the early stages of the pandemic, when most people were working at home.

Some impacts and underlying causes related to work/life balance identified in this study have been present since before the pandemic but were exacerbated as personal and professional lives became more intertwined when researchers were forced to work from home. With technical solutions installed and some experienced benefits of remote working, it is likely that it will become more common among RO researchers also after the pandemic. Nature’s fifth PhD survey in 2019 identified difficulty to maintain a healthy work/life balance as one of the most important sources of emotional strain for PhD students [49]. Further, it should be noted that the pandemic added to an already strained professional landscape. Reports of burn-out and low professional quality-of-life have been highlighted across all RO professions [50], [51], [52], [53], [54]. For this reason, we believe the insights and recommendations formulated in this study will remain important and helpful after the pandemic, to mitigate adverse consequences of working remotely and ensure sustainable development of the RO professional environment.

## Methodological considerations

This study focussed on (early-career) RO researchers working in European institutions and was aimed at capturing an extremely dynamic process in a limited timeframe. While a final oFG organised several months after the first three oFGs revealed a time and context dependency, no new insights were verbalised indicating data saturation was reached. Nevertheless, it is possible that certain experiences and scenarios were overlooked due to the finite number of participants and possible participation bias. In our study, we applied a convenience sampling strategy. This could mean that those most affected by the disrupted working conditions caused by COVID-19 did not take part, thereby biasing the overall results towards an impression of high self-efficacy that may not be true for most affected people. However, those most affected by the issues under study also tend to be the ones most willing to participate; they feel most connected with the topic and want to share their experiences and opinions [55]. Of note 50% of all volunteers were working in either The Netherlands or the United Kingdom, countries that are highly represented among ESTRO members, and the study attracted mostly female volunteers (81%). The gender disparity may be a manifestation of participation bias with women being generally more involved in supporting employee well-being compared to men [56]. However, the aim of the study was not to be exhaustive, but to gain a deeper understanding of the underlying mechanisms of how the consequences of disrupted working conditions (exemplified by the pandemic) arise and could be mitigated. We believe that with our strategy – where we aimed for diversity in our participants and for cross- validation with the organization of a fourth oFG – we were able to achieve this. The results of the cross-impact analysis represent the most probable impact scenarios, giving a more nuanced view on the topic compared to quantitative surveys. To the authors knowledge, this is one of only few qualitative research studies performed in the field of RO. Compared to quantitative analysis, qualitative research methods typically focus on exploring causal relations as opposed to identifying correlations, which can be of important value in other areas of RO research such as professional development and patient communication/education [57], [58], [59].

## Conclusion

In conclusion, this study has shown the highly individualized mechanisms and the diverse and dynamic interaction between identified variables in the context of disrupted and evolving working conditions, such as those experienced during the COVID-19 pandemic. To illustrate and analyse these complex dynamics, the impact model for disrupted working conditions has been developed. The model forms a framework to be used during discussions and reflections on work-life balance and burn-out prevention and can be used by teams, supervisors and institutions. We proposed recommendations and solutions to support early-career researchers, supervisors, and Heads of Department, as well as institutions and European or Global organizations in their efforts to maintain or improve a sustainable work/life balance for researchers in RO.

## Declaration of Competing Interest

The authors declare that they have no known competing financial interests or personal relationships that could have appeared to influence the work reported in this paper.

## Footnotes

3 Through a qualitative cross-impact analysis “impacting” and “impacted variables” are mapped. Impacting variables are the variables that have an impact on other variables. Impacted variables are the variables that impacting variables have an impact on. To improve readability, we use the terms “influential” and “affected variables” throughout the text.
