# Supplementary material for: An impact model to understand and improve work-life balance in early-career researchers in radiation oncology
Source: Clin Transl Radiat Oncol. 2022 Sep 26;37:101–8. doi: 10.1016/j.ctro.2022.09.006 (PMC9523090; doi:10.1016/j.ctro.2022.09.006)
Supplement: Supplementary data 1 [file mmc1.docx]

**Supplementary Materials**

**Illustrative quotes**

| Quote 1 | “*What was very difficult for me, is that I had to work from home with the kids. Because the schools were closed also during the first wave, the first lockdown, here in [country]. So that was really, very, very difficult and uhm, it was impossible to focus and work.*” (oFG3, participant 2) |
| --- | --- |
| Quote 2 | “*We have to do most of our research out of the working hours, during the weekends or something. And, so, since you couldn’t do that much, because everything was closed and you could not travel, you spend a lot of time at home. I also had much more time to do my research, and actually, I was a bit more productive than usually, I would say*.” (oFG2, participant 3) |
| Quote 3 | “*It is much harder to reach colleagues, I mean even if you don’t have to schedule meetings or things like…. I recognise that it takes a lot longer to reach colleagues and also, for brainstorming sessions if you have an idea that you just want to bounce of off people to help you along creatively.*” (oFG1, participant 6) |
| Quote 4 | *“Over the first lockdown it was actually quite nice to be at home. I got to start some hobbies that I didn’t have time for when I was always at the hospital. And, it was also coming in to summer at that time, so we were able to walk outside and do things that I didn’t have time when I was commuting all the time. But, over the winter period when the measures went on and on and on, it was very stressful at work and as I said we had a lot of people on mental health leave and I think that the other half of the team was very, very close and I definitely*  *experienced this myself as well.”* (oFG4, participant 6). |
| Quote 5 | *“I’m not sure if this [addressing mental health] is something a workplace should, but I think now maybe, during the pandemic, the workplace can like provide links to information or something so that you can make the own decision.”* (oFG3, participant 5) |
| Quote 6 | *“I think at the beginning you’re responsible of yourself and your projects, so I can understand that we need rules and we need support, but as I said, in this moment it is not possible. So, I think that you have to understand clearly by yourself what you want to do, what you can obtain and then you have to talk with your supervisor, your professor and your colleagues.”* (oFG4, participant 7) |
